# Comparison of the Mini Mental State Examination and depressive symptoms between high cardiovascular risk and healthy community elderly groups

**DOI:** 10.1590/S1980-57642009DN20400011

**Published:** 2008

**Authors:** Amanda Lucas da Costa, Juliana Santos Varela, Osmar Mazetti, Luciane Restelatto, Andry Fitterman Costa, Claudia Godinho, Ana Luiza Camozzato, Paulo D. Picon, Márcia L. Chaves

**Affiliations:** 1 Dementia Clinic, Neurology Service, Hospital de Clínicas de Porto Alegre.; 2 Medical Sciences Post-Graduation Course, UFRGS School of Medicine.; 3 Internal Medicine Department, UFRGS School of Medicine.; 4 Centro de Dislipidemia e Alto Risco Cardiovascular (CDA) from Hospital de Clinicas de Porto Alegre.

**Keywords:** cognitive disorder, cardiovascular risk, Mini Mental State Examination, depressive disorders

## Abstract

The aging of the population is a universal phenomenon with direct consequences upon the public health system. One of the main repercussions of the growth in this sector of the population is the increased prevalence of disorders such as dementia and depression which are very frequent among the elderly. The relationship between cardiovascular risk factors, dementia and depression have been addressed in many recent investigations.

**Objectives:**

To evaluate the relationship of cognitive performance and depressive symptoms with cardiovascular risk in the elderly.

**Methods:**

94 high cardiovascular risk elderly patients and 160 healthy community elderly were evaluated cross-sectionally. The Mini Mental State Examination (MMSE) and the Geriatric Depression Scale (GDS-15) were used as the main measures. The cutoff for presence of depression was 6 on the GDS.

**Results:**

The high cardiovascular risk elderly group showed significantly lower scores on the MMSE (p<0.001) and was significantly associated to depression (p<0.001), independently of education. The logistic regression analysis for depression as the dependent variable, age and group (healthy community or high cardiovascular risk elderly) were kept in the final equation. Higher age (Odds Ratio=0.92; 95% CI 0.86–0.98) and high cardiovascular risk elderly (OR=2.99; 95% CI 1.36–6.59) were associated to depression.

**Conclusions:**

The present findings corroborate the different cognitive performance of elderly with high cardiovascular risk factors and the association of depressive symptoms with this group.

## Abstract (pt)

O envelhecimento da população é um fenômeno mundial com conseqüências diretas no sistema de saúde pública. Uma das principais conseqüências do crescimento desta parcela da população é o aumento da prevalência de doenças como demência e depressão que são muito freqüentes entre os idosos. Recentemente, a relação entre fatores de risco cardiovasculares, depressão e demência foi abordada em várias investigações.

**Objetivos:**

Avaliar a relação de desempenho cognitivo e sintomas depressivos com risco cardiovascular em idosos.

**Métodos:**

94 idosos de alto risco cardiovascular e 160 idosos saudáveis da comunidade foram avaliados num corte transversal. O Mini-Exame do Estado Mental (MEEM) e a escala de depressão geriátrica (GDS-15) foram usados para as medidas principais. O ponto de corte para presença de sintomas depressivos foi 6 na GDS.

**Resultados:**

O grupo de alto risco cardiovascular mostrou escores significativamente mais baixos no MEEM (p<0001) independente da educação, e foi significativamente associado a depressão (p<0,001). A análise de regressão logística para depressão como variável dependente, idade e grupo (idosos saudáveis da comunidade ou idosos de alto risco cardiovascular) foram mantidos na equação final. Maior idade (Razão de Chance=0,92, IC 95% 0,86–0,98) e idosos de alto risco cardiovascular (RC=2,99; IC 95% 1,36–6,59) estavam associados à presença de depressão.

**Conclusões:**

Os achados do presente estudo corroboram o desempenho cognitivo diferencial dos idosos de alto risco cardiovascular e a associação de sintomas depressivos a este grupo.

The aging of the population is a universal phenomenon with direct consequences upon the public health system. As life expectancy increases, the number of demented patients worldwide is projected to grow from 24.3 million in 2001 to 81.1 million by 2040.^1^ Approximately 6 million Americans and 2 million Brazilians have Alzheimer’s disease.^2,3^ AD is the 3rd most expensive disease in USA, after heart disease and cancer, and is among the 10 leading causes of death among people >65 years’ old in developed countries.^2^ Therefore, the identification of individuals at risk for developing dementia is essential. Early diagnosis allows therapeutic interventions, decreases families’ level of stress, reduces incidental risk, increases autonomy and maybe in some cases prevents or retards dementia onset.^4^

Since depression and dementia are among the most common mental health problems in the elderly population, it is common to observe their comorbid presentation, which impacts quality of life, functional decline, increase in the use of health services, increase of morbidity and mortality.^5^ Depression is probably the most frequent cause of emotional distress and worsening of life quality among the elderly.^6,7^ This population is more prone to developing depression due to reduced social perspectives, health impo­v­erish­ment, frequent losses, biological, vascular, structural and functional changes, besides neuroendocrine and neurochemistry dysfunction in the brain during aging.^8^ Therefore, development of elderly depression is multi-factorial in nature.^5^ The association between depression and dementia is also significant. Depressed elderly may present cognitive decline, besides an increased risk for progression to dementia.^9^

Over the last 3 decades, a decline of cardiovascular-related mortality has been observed in developed countries, while relatively fast and substantial increases have occurred in developing regions, such as Brazil. According to the World Health Organization, the tendency for increase among developing areas may persist, worsening the scenario of already high morbidity and mortality.^10^ The higher risk for developing dementia is found among patients with conditions associated to increased cholesterol levels, such as cardiovascular diseases and atherosclerosis.^11^

Studies have been carried out seeking to better understand the role of atherosclerotic disease, dyslipidemia and hypertension, predictive factors of cardiovascular risk, in the development of cognitive deficit. The strong association between atherosclerosis and dementia, especially Alzheimer’s disease, has previously been demonstrated. Patients with vascular disease have shown three-fold higher risk for developing dementia.^12^ Prospective studies have shown the relationship of atherosclerotic disease and cognitive decline among the elderly. It is presumed that atherosclerosis may cause neuronal damage through ischemic lesions, followed by inflammatory response that leads to neuronal degeneration. Another hypothesis is neuronal damage caused by hypoperfusion due to decreased blood flow in the brain.^13^ A higher frequency of brain ischemic events was found in dementia patients compared to non-demented individuals. In these patients, the observation of atherosclerotic plaques in major vessels presented strong correlation with deposition of neuritic plaques, whose main component is the B-amyloid protein associated to Alzheimer’s disease.^14^ However, no association between generalized atherosclerosis and depression in the elderly was observed.^13^

The link between dyslipidemia and the occurrence of cognitive deficit has been previously evaluated. The role of higher levels of cholesterol was significant as a risk factor for dementia in middle-age patients, but not among elderly patients.^12^ The substantially lower risk of cognitive decline was demonstrated in patients over the age of 50 with the use of statins^15^. Adult hypertension increases the risk of dementia, especially when associated to hypercholesterolemia.^16^

Hypertension is associated to increased risk of dementia, and control with anti-hypertensives, mainly with angiotensin converting enzyme inhibitors, has demonstrated a significant reduction in the incidence of dementia.^12^ Diabetes is an independent predictor of risk of cognitive decline, and the metabolic syndrome, an important cardiovascular risk factor, plays a role as a predictor of risk for dementia.^17^

The association of depression to cardiovascular disease is based on convincing evidence. A bidirectional pathway was proposed for these diseases because depression is an independent risk factor for cardiovascular events, and is more prevalent in patients with these conditions. Depression aggravates vascular disease and is associated to poorer outcomes.^18^ The association between risk for cardiovascular events, and cognitive decline and depressive symptoms make screening in this population imperative.

The present study aimed to evaluate the relationship of cognitive performance and depressive symptoms with cardiovascular risk in the elderly. The performance on the Mini Mental State Examination and the Geriatric Depression Scale among a sample of dyslipidemic elderly outpatients with high cardiovascular risk and a sample of healthy community elderly was compared.

## Methods

A cross-sectional study was carried out for the objectives under investigation. The sample was composed of 94 elderly recruited from the reference center for dyslipidemia and high cardiovascular risk (Centro de Dislipidemia e Alto Risco Cardiovascular) from Hospital de Clínicas de Porto Alegre along with 160 healthy community elderly drawn from a cohort within the catchment area of the same hospital. All participants were aged 60 years or more. Illiteracy was not an exclusion criterion for either of the groups.

All patients evaluated at the reference center for dyslipidemia and high cardiovascular risk underwent a detailed clinical examination with an emphasis on past or recent cardiovascular events, physical and neurological examination and laboratory tests. Total cholesterol, HDL cholesterol, LDL cholesterol, triglycerides, glucose, ALT, AST, GGTP, electrolytes, creatinine, uric acid and TSH were the blood tests evaluated.

The inclusion criteria for dyslipidemia among the high cardiovascular risk group were based on the clinical guidelines for treatment with statins for this disease developed by the Brazilian Health Ministry.^19^ One of the three following criteria (a, b or c) had to be met for inclusion:

- cholesterol LDL>100mg/dl plus acute myocardial infarction;
- cholesterol LDL>130mg/dl plus one of the following situations: coronary heart disease evidenced by exercise testing or myocardial scintigraphy, previous myocardial infarction, coronary revascularization, or at least 30% obstruction on coronary angiography; other atherosclerotic disease such as peripheral vascular disease with intermittent claudication or obstruction ≥50% on Doppler, aortic abdominal aneurysm and/or symptomatic carotid disease; diabetes mellitus; genetic syndromes – familial hypercholesterolemia and lipid disorder; higher Framingham score (≥9 for men and ≥15 for women);
- cholesterol LDL>160mg/dl plus one of the following situations: Framingham score (≥6 for men and =10 for women); age ≥55 years; hypertension; HDL <40 mg/dl; cigarette smoking.

Patients with abnormal neurological examination or previous history of stroke and dementia were excluded from this group. All selected patients were using statins regularly.

The group of healthy community elderly was drawn from an ongoing cohort study.^20^ All selected participants fulfilled criteria for the healthy aging study and consented to participate. The inclusion and exclusion criteria are depicted in Table 1. Briefly, participants underwent a standardized neuropsychological and neurological evaluation. A collateral informant was also used to verify the history. Subjects were excluded if they had age-related diseases or risk factors for cognitive impairment at baseline. All participants and their collateral informants had to report normal functioning in the community at study entry and were screened with the Clinical Dementia Rating scale.^21,22^ Participants with a CDR of 0.5 (suggestive of incipient dementia) or greater (suggestive of dementia) were excluded from the sample.

**Table 1:** Participant selection criteria for the elderly community cohort.

Requirements for entry	Major exclusion criteria
Functionally independent	Medical conditions
Gives informed consent	Myocardial infarction
Willing to participate in the follow-up	Diabetes mellitus
Score=0 on Clinical Dementia Rating Scale	Chronic pulmonary disorder
Score >11 on the Blessed	Chronic renal disease
Information-Memory-Concentration Test	Hypertension (supine blood pressure >160/95)
	Active cancer
	Seizure disorder
	Stroke/transient ischemic attack
	Parkinson disease
	Other neurological disorders (LAS, MS, etc.)
	Major Surgeries
	Coronary bypass
	Carotid endarterectomy
	Psychiatric conditions (previously diagnosed)
	Schizophrenia
	Major affective disorder
	Phobias
	Chronic anxiety
	Alcohol or drug abuse
	Vision and Hearing
	Vision uncorrectable to 20/100 OU
	Hearing loss (interferes with speech perception)
	Other conditions
	Significant head injury
	Unexplained prolonged loss of consciousness
	Use of medications impairing cognitive function

Demographic data, the Mini-Mental State Examination^23-25^ and the Geriatric Depression Scale (GDS-15)^26^ were assessed in both groups. Subjects were further categorized into those with or without depressive symptoms according to the cutoff of 6 on the GDS^29^

The study was approved by the Ethics Committee for Research of the Hospital de Clínicas de Porto Alegre. All subjects signed an informed consent.

### Data analysis

The statistical analysis was performed by the *Statistical Package for the Social Sciences* (SPSS for Windows 13.0) software. Parametric variables were analyzed with Student’s t test. The Chi-square test (with Yates correction or Fisher exact) was used for the association analysis. One-way ANOVA with covariance analysis and the logistic regression model were used as multivariate models.

## Results

Comparison of demographic, MMSE score and presence or absence of depressive symptoms by GDS between the dyslipidemic patients with high cardiovascular risk and the community healthy elderly groups are displayed in Table 2. Education was significantly higher in the healthy community elderly group (p<0.001). The dyslipidemia with high cardiovascular risk elderly group showed significantly lower scores on the MMSE (p<0.001) and was significantly associated to depressive symptoms (p<0.001). On one-way ANOVA with covariance, group (p<0.001) and educational level (p<0.001) showed independent and significant effects on MMSE scores.

**Table 2:** Demographic and clinical data of high cardiovascular risk group and healthy community elderly group.

Variables	High cardiovascular risk elderly with dyslipidemia (n=94)	Healthy community elderly (n=160)	p
Age (mean ± SD)*	66.3±6.2	67.7±5.8	0.062
Sex**			
Female (N %)	60 (63.8%)	108 (67.5%)	0.551
Education (mean ± SD)*	2.4±6.49	6.49±3.39	<0.001
MMSE (mean ± SD)*	24.24±5.28	25.46±3.22	<0.001
GDS (depression)**			
Yes (N %)	31 (33%)	18 (11.3%)	<0.001

* Student t test;

** Chi-square association test.

In the multivariate analysis (logistic regression) with depression as the dependent variable, age and group (healthy community or dyslipidemia with high cardiovascular risk elderly) were kept in the final equation (Table 3). Higher age (Odds Ratio=0.92; 95% CI 0.86–0.98) and dyslipidemia among the high cardiovascular risk elderly group (OR=2.99; 95% CI 1.36–6.59) were associated to depression.

**Table 3:** Logistic regression for depression outcome with age, group (healthy elderly or high cardiovascular risk elderly with dyslipidemia), education, MMSE and sex as independent variables.

Variables	B	Wald	p	OR (95% CI)
Group*	1.096	7.403	.007	2.99 (1.36-6.59)
Education	-.044	.513	.474	0.96 (0.85-1.08)
Age	-.083	6.693	.010	0.92 (0.86-0.98)
MMSE	-.056	1.643	.200	0.95 (0.87-1.03)
Sex	-.570	2.071	.150	0.56 (0.26-1.23)
Constant	5.291	4.234	.040	198.514

* High cardiovascular risk elderly with dyslipidemia was the reference group.

## Discussion

This study was carried out to analyze both cognitive performance measured by the Mini Mental State Examination (MMSE), and depressive symptoms using the Geriatric Depression scale (GDS) in high cardiovascular risk elderly with dyslipidemia and to compare results with healthy community elderly. We observed lower MMSE scores among the dyslipidemia with high cardiovascular risk elderly as well as greater depression in this group, independently of educational level. Vascular risk factors may impair cognitive functions and are related to the occurrence of not only vascular dementia but also Alzheimer’s disease.^12^ The level of evidence for these associations is highest for hypertension and diabetes mellitus(DM), especially when these factors are assessed in middle age.^17,27-29^ However, the essential pathophysiological mechanisms were still not linked to clinical relevance.^12^ Several studies have highlighted the possible protective effect of antihypertensive therapy on cognition while some trials are assessing the effects of statins and treatments for insulin-resistance.^15,30-33^

In the logistic regression for depression controlled for education, both higher age and high cardiovascular risk elderly were risk factors. Elderly with dyslipidemia with high cardiovascular risk presented twice the risk of presenting depressive symptoms than healthy community elderly from the same age group.

Emerging evidence has suggested a causal relationship between atherosclerosis and both cognitive decline and depression in old age.^34,35^ In addition, neuropathologic findings indicate that subjects with cognitive impairment more often have vascular pathology^36,37^, whereas late-life depression has been associated with white matter hyperintensity on brain neuroimaging, assumed to be vascular in origin.^18,38,39^ Recognition of the relationship between cerebrovascular disease and depressive symptoms has led to the proposition of the term “vascular depression” to describe a clinical subtype of major depression characterized by apathy, psychomotor changes, and cognitive impairment in the presence of cerebrovascular disease demonstrated on neuroimaging or by focal neurological findings (e.g., mild hemiplegia, facial droop).^9,40^

Considering that cognitive decline, dementia and depressive symptoms are a worldwide problem, establishing preventive or curative treatment when available is a major health challenge, and vascular risk factors are a promising research pathway for these conditions.
